# Genetic architecture of seedling stage chilling tolerance in United States rice germplasm

**DOI:** 10.3389/fpls.2026.1845537

**Published:** 2026-07-09

**Authors:** Raveendra Chandavarapu, Renganathan Vellaichamy Gandhimeyyan, Brijesh Angira, K. Raja Reddy, Prasanta K. Subudhi, Raju Bheemanahalli

**Affiliations:** 1 Department of Plant and Soil Sciences, Mississippi State University, Mississippi State, MS, United States; 2 Louisiana State University Agricultural Center, H. Rouse Caffey Rice Research Station, Rayne, LA, United States; 3 School of Plant, Environmental, and Soil Sciences, Louisiana State University Agricultural Center, Baton Rouge, LA, United States

**Keywords:** candidate genes, chilling stress, GWAS, rice, seedling stage, stress tolerance indices

## Abstract

Chilling stress during the seedling stage severely limits rice growth and development, highlighting the need for a deeper understanding of the genetic architecture underlying chilling stress tolerance. In this study, a rice association panel was evaluated for seedling vigor traits under growth-chamber conditions (Experiment 1) and under natural chilling conditions (Experiment 2). Nine stress tolerance indices (STIs) were calculated from Experiment 1 for the seedling traits. Genome-wide association studies (GWAS) were conducted using 872,995 high-density single-nucleotide polymorphisms (SNPs) and four models, including two single-locus (GLM and MLM) and two multi-locus (FarmCPU and BLINK) models. A total of 54 quantitative trait loci (QTLs) associated with seedling morphological traits were identified, with phenotypic variance explained (PVE) ranging from 5.3 to 82.2%. Twenty-eight QTLs were co-localized with previously reported loci, confirming the reliability of the associations, while one novel QTL, *qNL2-1*, was consistently detected in both Experiment 1 and STI-based analyses. Candidate gene mining within the identified QTL regions revealed several putative genes involved in calcium signaling, transcriptional regulation, and stress response pathways, including calmodulin (CaM)/CaM-like (CML) proteins, calcium-dependent protein kinases (CDPKs), calcineurin B-like (CBL) proteins, CBL-interacting protein kinases (CIPK), and brassinosteroid (BR) signaling proteins. The candidate genes identified in this study could serve as potential targets for enhancing rice chilling tolerance through genomics-assisted breeding strategies.

## 1 Introduction

Rice (*Oryza sativa* L.) is a staple food for half the world’s population. While it originated and initially cultivated in tropical regions (Cruz et al., 2013), it is now cultivated in over 100 countries, adapting to a variety of ecological conditions. However, this adaptability exposes rice to numerous abiotic stresses. With low temperature or chilling being a significant constraint to rice productivity in at least 25 countries (Cruz et al., 2013), affecting more than 15 million hectares globally (Shi et al., 2023). Chilling stress poses challenges in direct-seeded rice systems and in high-altitude tropical or temperate regions during germination and early growth and establishment stages, hindering plant stand density that translates into lower yield (Schläppi et al., 2017).

Chilling stress (<15 °C) during germination can reduce germination rates, delay emergence, and impair seedling establishment, ultimately leading to substantial yield losses (Viana et al., 2024). Typical symptoms of chilling-induced stress during the seedling stage include delayed germination, reduced germination percentage, leaf yellowing, wilting, and withering (Suzuki et al., 2008). During the vegetative phase, chilling inhibits photosynthetic activity by limiting electron transport in photosystems (Weon Jeong et al., 2002), causing energy imbalances and damage to photosynthetic proteins, thereby impairing ATP and NADPH production (Hüner et al., 2012). These energy imbalances promote the formation of reactive oxygen species (ROS), such as singlet oxygen, hydroxyl radicals, hydrogen peroxide, and superoxide anions (Foyer and Noctor, 2005). These effects are further exacerbated under intense light, inducing photoinhibition of photosystem II (PSII) in chilling-sensitive plants (Lukatkin, 2005). To counteract this damage, plants activate antioxidant enzymes, such as ascorbate peroxidase, superoxide dismutase, and catalase, to protect cells by scavenging ROS (Mittler, 2002).

Enhancing seedling survival and vigor under low temperatures is crucial for improving productivity in challenging environments, particularly through breeding temperature-tolerant varieties. Early vigor is important for canopy development and necessary for greater light interception, while poor root vigor can negatively impact later growth stages. Despite this, earlier studies on chilling tolerance in rice have primarily focused on scoring shoot traits, often overlooking the role of root traits. Notably, during early spring, the root zone may experience low air or soil temperatures due to the heat-conducting properties of water and soil, even as shoot temperatures are warmer (Ahamed et al., 2012). Such chilling conditions hinder root water uptake due to reduced root hydraulic conductivity (Lpr), thereby affecting plant water status, photosynthesis, and stomatal conductance (Kramer and Boyer, 1995; Yamori et al., 2010). Research shows that rice Lpr declines at temperatures below 15 °C (Murai-Hatano et al., 2008), whereas chilling-tolerant species exhibit increased Lpr (Bigot and Boucaud, 2000). Understanding the effects of chilling on early shoot and root vigor is crucial for breeding resilient rice cultivars. On the other hand, although *japonica* rice is generally believed to have greater cold tolerance than *indica* rice, the genetic basis of this adaptability remains poorly understood (Pan et al., 2015).

Quantitative Trait Locus (QTL) mapping and genome-wide association studies (GWAS) are two widely used approaches for identifying genetic regions associated with complex traits (Korte and Farlow, 2013). To date, most genetic studies of chilling tolerance in rice have been derived from biparental mapping populations, typically involving crosses between *indica* and *japonica* subspecies, with *japonica* often serving as the donor for cold tolerance (Ma et al., 2015). However, the limited genetic diversity in the bi-parental mapping population restricts the discovery of novel loci. In contrast, recent advances in GWAS have enabled more detailed investigations of the genetic architecture of key traits in rice. Early GWAS in rice by Huang et al. (2010) identified several genetic loci associated with grain yield and other agronomic traits, highlighting the potential of this approach to complement traditional linkage mapping. Since then, several studies have identified QTLs associated with cold tolerance in rice. For instance, Pan et al. (2015) identified 22 loci associated with cold tolerance for germination trait using 174 Chinese rice accessions. Fujino et al. (2015) mapped 23 QTLs related to heading stage (six QTLs) and low temperature germinability (17 QTLs) using a Hokkaido rice core panel. Lv et al. (2016) identified 132 loci across 16 traits in 529 accessions under chilling and cold shock conditions. Wang et al. (2016) identified 67 QTLs for seedling-stage cold tolerance across 295 genotypes. Sales et al. (2017) reported 24 SNPs associated with low-temperature germination and growth rates, while Shakiba et al. (2017) identified 42 QTLs for cold tolerance in seedlings. Schläppi et al. (2017) identified 48 QTLs associated with chilling tolerance across 202 accessions from the USDA mini-core collection. Collectively, these studies demonstrated associations between multiple genomic regions and cold tolerance, including the characterization of cold-related genes such as *OsSAP16* (Wang et al., 2018), *OsCTB2* (Li et al., 2021), and *OsCOLD11* (Li et al., 2025).

In the present study, a novel rice association panel was evaluated, and genomic regions associated with chilling tolerance were identified using high-density SNP markers. The specific objectives of this study were to (i) determine chilling-induced variations in shoot and root traits under controlled environment growth chamber (Experiment 1) and natural chilling (Experiment 2) conditions, (ii) identify genomic regions and candidate genes associated with chilling stress tolerance, and (iii) determine which stress tolerance indices (STIs) are best suited for discovering genetic loci associated with chilling stress tolerance. We anticipate that the identified chilling-tolerant accessions and loci will provide valuable resources for breeding rice cultivars with improved chilling stress tolerance and early vigor.

## 2 Materials and methods

### 2.1 Plant materials and growth conditions

The rice association panel consists of 233 accessions, mainly *japonica* (159), followed by *indica* (38) and admixture (36), and seeds were obtained from the LSU Agricultural Center (**Supplementary Table 1**). The panel comprises modern Southern US rice breeding germplasm, including released cultivars and advanced breeding lines from Louisiana, Arkansas, Texas, Mississippi, California, and Missouri (Angira et al., 2019).

### 2.2 Experiment 1: controlled environment growth chamber

Experiment 1 was conducted in controlled-environment growth chambers (Percival Scientific Inc., USA) at the R.R. Foil Plant Science Research Center, Mississippi State University, Mississippi, USA. Seeds were planted in 98-well plastic trays (60 × 30 × 16.8 cm) filled with a 1:1 soil: sand mixture, using a completely randomized design (CRD). Two batches were sown, each with four replications. Immediately after sowing, both batches were maintained in growth chambers at 30/22 °C (day/night), with a 14 h photoperiod (800 µmol m^-2^ s^-1^ light intensity), and 70% relative humidity for 14 days. At 14 days after sowing (two-leaf stage), two temperature regimes simulating Southern U.S. early planting (March) vs. optimum planting (April) conditions were imposed to represent “chilling” and “control” treatments, respectively. One batch of trays was maintained at 30/22 °C and served as the control (Environment 1, E1), while the temperature of the second batch was adjusted to 22/14 °C to impose chilling stress (Environment 2, E2). Temperature data were recorded throughout the experimental period (**Supplementary Figure 1**). Plants were harvested 14 days after the initiation of stress treatment to measure above-ground traits [number of leaves (NL), shoot length (SL), shoot biomass (SDW)] and below-ground [root length (RL), root biomass (RDW), and root-to-shoot ratio (R/S)] traits at the early vegetative stage.

### 2.3 Experiment 2: natural chilling

**Figure 1:**
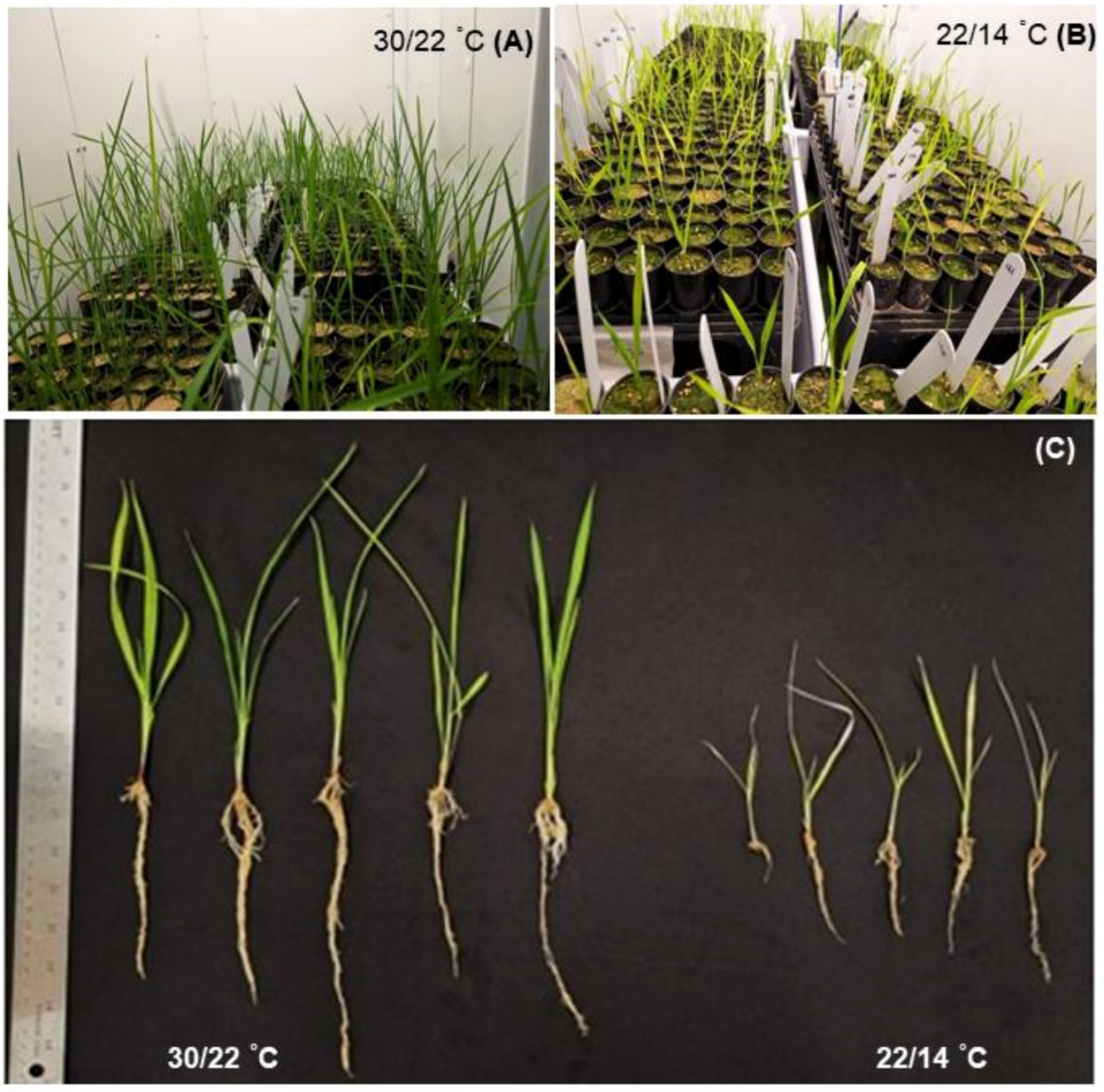
Overview of rice genotypes tested for seedling stage chilling stress in Experiment 1. Twenty-eight-day-old seedlings under **(A)** control (30/22 °C, day/night temperature), and **(B)** chilling (22/14 °C, day/night temperature) treatments. **(C)** Morphology of rice genotypes in control (left) and chilling in Experiment 1 (right).

Experiment 2 was conducted outdoors at the R.R. Foil Plant Science Research Center, Mississippi State University, MS, USA, from mid-March 2023. The same set of genotypes used in Experiment 1 was used for the outdoor study. As in Experiment 1, seeds were sown in plastic cones filled with a 1:1 mixture of soil and sand, placed in 98-well plastic trays, and maintained under outdoor conditions. Each genotype was replicated 5 times and grown under outdoor conditions or natural chilling (Environment 3, E3) during emergence and seed establishment. The average temperature during the experimental period was 15 °C; however, extreme temperatures ranging from 5 °C to 35 °C (night to daytime) were recorded on some days (**Supplementary Figure 1**). Soil temperature was recorded at 15-minute intervals using HOBO data loggers (Onset Computer Corp., MA, USA) placed at a depth of 5 cm in the soil within the 98-well plastic tray. Trait values in E3 were normalized to E1, which represents typical agronomic conditions for early April planting in the Southern US. Although both treatments (E1 and E3) differed in certain environmental factors, E1 served as the baseline for comparison. Due to inherent limitations of field conditions, establishing a true field-based control that isolates the effect of temperature is not feasible under natural chilling conditions. The experimental setup and representative morphological observations of rice seedlings are shown in **Figure 1**.

### 2.4 Stress tolerance index

In earlier studies, several stress-tolerance indices (STIs) have been used to screen for and identify tolerant accessions or genetic loci under stress. However, no studies have examined whether all stress tolerance indices (STIs) for a given trait detect the same genetic loci under specified stress conditions. In this study, phenotypic data from E1 and E2 of Experiment 1 were used to calculate various STIs using the formulas described below, and GWAS was performed for each calculated index.

Stress tolerance (ST) = Yp – Ys (Rosielle and Hamblin, 1981)

Stress tolerance index (STI) = (Yp × Ys)/Xp^2^ (Fernandez, 1992)

Relative stress index (RSI) = (Yp/Ys)/(Xs/Xp) (Fischer and Wood, 1979)

Mean relative performance (MRP) = (Ys/Xs) + (Yp/Xp) (Ramirez-Vallejo and Kelly, 1998)

Stress susceptibility index (SSI) = 1-(Ys/Yp)/1-(Xs/Xp) (Fischer and Maurer, 1978)

Mean productivity (MP) = (Yp + Ys)/2

Abiotic tolerance index (ATI) = [(Yp - Ys)/(Xp/Xs)] × 100 (Moosavi et al., 2008; Kalve et al., 2022)

Stress susceptibility percentage index (SSPI) = [(Yp - Ys)/(2Xp)] × 100 (Moosavi et al., 2008)

Modified stress tolerance index (MSTI) = (Yp^2^/Xp^2^) × [(Yp + Ys)/Xp^2^] (Farshadfar and Sutka, 2002)

Where Yp, Ys, Xp, and Xs represent the performance of individual accessions in a non-stress environment, the performance of the individual accessions in a stress environment, the average performance of all accessions in the non-stress environment, and the average performance of all accessions in the stress environment, respectively. In addition, a ranking approach based on STIs was used to identify superior chilling-tolerant accessions by calculating the mean rank of each accession across all indices. Phenotypic data from Experiments 1 and 2 and the derived STIs were subsequently used in GWAS to identify significant genomic regions associated with chilling stress tolerance.

### 2.5 DNA extraction and sequencing

Leaf tissues from 233 rice accessions were collected at the two-leaf stage and shipped to the Institute of Biotechnology at Cornell University for DNA extraction, library preparation, and sequencing. Skim sequencing (skim-seq) libraries were generated using the Nextera DNA library preparation protocol (Illumina Inc., San Diego, CA, USA), which employs tagmentation. In this process, a transposome complex (comprising a transposase and a transposon) simultaneously induces double-strand breaks in genomic DNA and ligates sequencing adapters in a single step. One library was prepared per sample, and 1 µL from each library was pooled to initiate sequencing. The pooled library underwent an initial size selection using the BluePippin system (Sage Science, Beverly, MA, USA), targeting DNA fragments between 450 and 1000 base pairs (bp) to improve sequencing uniformity. Initial sequencing was performed on an Illumina MiSeq Nano platform using paired-end (2 × 150 bp) sequencing, yielding approximately 1 million reads per library. Libraries were subsequently re-normalized by read counts and re-pooled to ensure balanced representation in high-throughput sequencing. A second round of size selection using the BluePippin system was performed to further refine the fragment size distribution. Final sequencing and genotyping were conducted at Weill Cornell Medicine using the Illumina NovaSeq 6000 platform. Paired-end sequencing (2 × 150 bp) generated approximately 400 to 500 gigabase pairs of data, corresponding to an average genome coverage of 2-3X per sample. Raw data comprising 4.7 million SNPs distributed across 12 chromosomes were obtained after mapping to Nipponbare reference coordinates.

### 2.6 Genome-wide association study

The raw SNPs dataset was further subjected to quality control using TASSEL 5.2 software (Bradbury et al., 2007). SNPs with a missing rate exceeding 10% and minor allele frequency (MAF) below 5% were removed, and missing genotypes were imputed using the LD-kNNi method implemented in TASSEL 5.2 (Money et al., 2015). After filtering and imputation, 872,995 SNPs were retained for GWAS. Genome-wide association analyses were performed using the Genome Association and Prediction Integrated Tool (GAPIT: version 3), with two univariate (GLM: General Linear Model and MLM: Mixed Linear Model) and two multivariate models (FarmCPU: Fixed and random model Circulating Probability Unification and BLINK: Bayesian-information and Linkage-disequilibrium Iteratively Nested Keyway) (Lipka et al., 2012). The GLM incorporated principal components analysis (PCA) to account for population structure, while the MLM included a kinship matrix (K) to correct family-relatedness and reduce false-positive associations. BLINK and FarmCPU are computationally efficient approaches that leverage linkage disequilibrium (LD) information and pseudo-quantitative trait nucleotides, respectively, to reduce false negatives observed in MLM-based analyses. The LD between SNPs was estimated using the squared allele frequency correlation (r²) in TASSEL 5.2. The genome-wide average maximum r^2^ value was 3.71, which declined to 1.85 at a physical distance of 673,984 base pairs (bp), corresponding to the estimated LD decay distance. Quantile–quantile (Q–Q) plots were generated to compare observed and expected p-values to assess the control of type I errors (false positives). The significance threshold for SNP-trait associations was determined using the Bonferroni correction, calculated as -log10(0.01/872,995) ≈ 7.94. Putative candidate genes were identified based on the following criteria: (1) a QTL region was defined as a 670 kb interval centered on the significant SNP, corresponding to the estimated LD decay distance, and (2) the SNP was detected by at least two of the four models (GLM, MLM, FarmCPU, BLINK). Functional annotations of genes within QTL regions were retrieved from the Rice Annotation Project Database (RAP-DB) (Sakai et al., 2013). Identified regions were compared with previously reported QTLs using the Q-TARO (QTL Annotation Rice Online) database and published literature. In addition, the expression patterns of the putative candidate genes in different tissues, including leaves, shoots, and roots, were obtained from the Plant Public RNA-seq Database (Yu et al., 2022).

### 2.7 Data analysis

For both experiments, all the traits were subjected to analysis of variance (ANOVA) using the following linear model in R v4.2.2 software (R Core Team, 2023).


$${\text{Y}}_{\text{ij}}=\mu +{\text{T}}_{\text{i}}+{\text{E}}_{\text{ij}}$$

Where Y_ij_ is the response variable, μ is the overall mean, T_i_ is the ith treatment effect, and E_ij_ is the random error. A Pearson correlation matrix was prepared using the ‘GGally’ (Schloerke et al., 2024) and ‘metan’ (Olivoto and Lúcio, 2020) packages in R. Frequency distributions and mean ranking across the stress tolerance indices were calculated using ‘psych’ and ‘dplyr’ packages in R (Revelle, 2023). The PVE for each locus is calculated using the following formula (Zhang et al., 2019).


$$\text{PVE }\left(\text{Vpi}\right)=\frac{2qi\left(1-qi\right)\beta^{2}i}{\sigma^{2}p}\times 100$$

Where qi is the minor allele frequency of *i*th SNP, βi is the effect of *i*th SNP, and *σ*^2^*p* is the phenotypic variance of trait.

## 3 Results

### 3.1 Phenotypic response to seedling-stage chilling stress

ANOVA showed significant differences (p < 0.01) among accessions in NL, SL, RL, SDW, RDW, and R/S in response to chilling stress. Descriptive statistics for traits measured in Experiment 1 (E1 and E2) and Experiment 2 (E3) are presented in **Table 1**. The panel exhibited high genetic diversity, supporting its suitability for GWAS (**Table 1**). The mean NL values were 4.3 (E1), 2.8 (E2), and 3.7 (E3). Shoot length (SL) decreased under chilling stress, averaging 21.0 cm in E1, 13.4 cm in E2, and 10.8 cm in E3. In contrast, root length (RL) increased under chilling stress, with mean values of 12.4 cm (E1), 11.6 cm (E2), and 16.3 cm (E3). Shoot dry weight (SDW) declined substantially under chilling stress, with means of 52.5 g (E1), 22.5 g (E2) and 27.1 g (E3), while root dry weight (RDW) followed a similar trend (24.2 g in E2, and 23.0 g in E3). The root-to-shoot ratio (R/S) increased under chilling conditions, with values of 0.4 (E1), 0.5 (E2) and 0.8 (E3) (**Table 1**). Overall, NL, SL, SDW, and RDW in E2 were reduced by 34%, 36%, 57%, and 52%, respectively, while R/S increased by 25% relative to E1. Under natural chilling (E3), NL, SL, and SDW decreased by 14%, 48%, and 48%, respectively, while RL and R/S increased by 31% and 100% relative to E1. A noteworthy observation was that, although accessions produced very short shoots in E3, they developed a comparatively large root system (RL and R/S) under natural chilling (E3), a pattern not observed under E1 conditions. The phenotypic analysis clearly indicates that chilling stress significantly suppresses above-ground traits while promoting a shift in biomass allocation toward the root system, as reflected by increased R/S in both E2 (0.5) and E3 (0.8) compared to the control, E1 (0.4). Stress tolerance indices (STIs) from Experiment 1 and Experiment 2 for the 233 accessions (based on mean NL, SL, RL, SDW, RDW, and R/S) are summarized in **Supplementary Table 2** (all accessions) and **Supplementary Table 3** (top 20 chilling-tolerant accessions). In addition, genetic variability parameters, including the genotypic and phenotypic coefficients of variation, heritability, and genetic advance as a percentage of the mean, were calculated for both experiments (**Supplementary Figure 2**).

**Table 1:** Descriptive statistics of seedling traits in 233 rice genotypes in the control and chilling treatments.

Trait (unit)	Treatment	Min	Max	Mean	SD	CV
Number of leaves (NL)	Environment 1	2.8	7.0	4.3	0.7	17.0
	Environment 2	1.0	4.5	2.8	0.6	22.0
	Environment 3	1.0	5.0	3.7	0.6	16.7
Shoot length (SL, cm)	Environment 1	11.0	36.0	21.0	5.0	23.5
	Environment 2	6.7	19.2	13.4	2.2	16.7
	Environment 3	3.0	16.6	10.8	2.3	21.7
Root length (RL, cm)	Environment 1	2.5	23.0	12.4	3.5	27.7
	Environment 2	2.5	34.4	11.6	3.4	29.5
	Environment 3	2.7	24.0	16.3	3.1	18.7
Shoot biomass (SDW, g plant^-1^)	Environment 1	13.5	126.6	52.5	22.1	42.2
	Environment 2	5.3	50.2	22.5	7.9	35.3
	Environment 3	1.9	58.5	27.1	10.6	46.0
Root biomass (RDW, g plant^-1^)	Environment 1	3.5	73.8	24.2	13.9	57.7
	Environment 2	1.6	29.6	11.5	4.7	40.8
	Environment 3	0.8	55.6	23.0	10.3	38.0
Root-to-shoot ratio (R/S)	Environment 1	0.2	0.9	0.4	0.1	27.4
	Environment 2	0.1	1.1	0.5	0.1	27.4
	Environment 3	0.1	2.2	0.8	0.3	31.6

SD, Standard deviation; CV, Coefficient of variation.

### 3.2 Pearson’s correlation analysis

**Figure 2:**
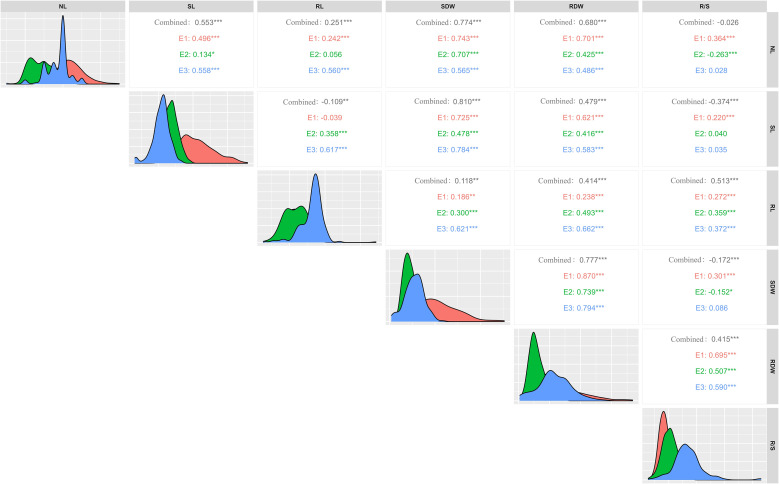
Pearson’s correlation coefficients among the traits. Environment 1 (E1, Control); Environment 2 (E2, Chilling); Environment 3 (E3, Natural chilling). E1 and E2 are growth chamber studies in experiment 1. NL: Number of leaves; SL: Shoot length; RL: Root length; SDW: Shoot biomass; RDW: Root biomass; R/S: Root-to-shoot ratio. Red, Green, and Blue colors represent E1, E2, and E3, respectively. Combined – indicates correlation values using the combined dataset of E1, E2, and E3. *p < 0.05; ***p < 0.001.

**Figure 3:**
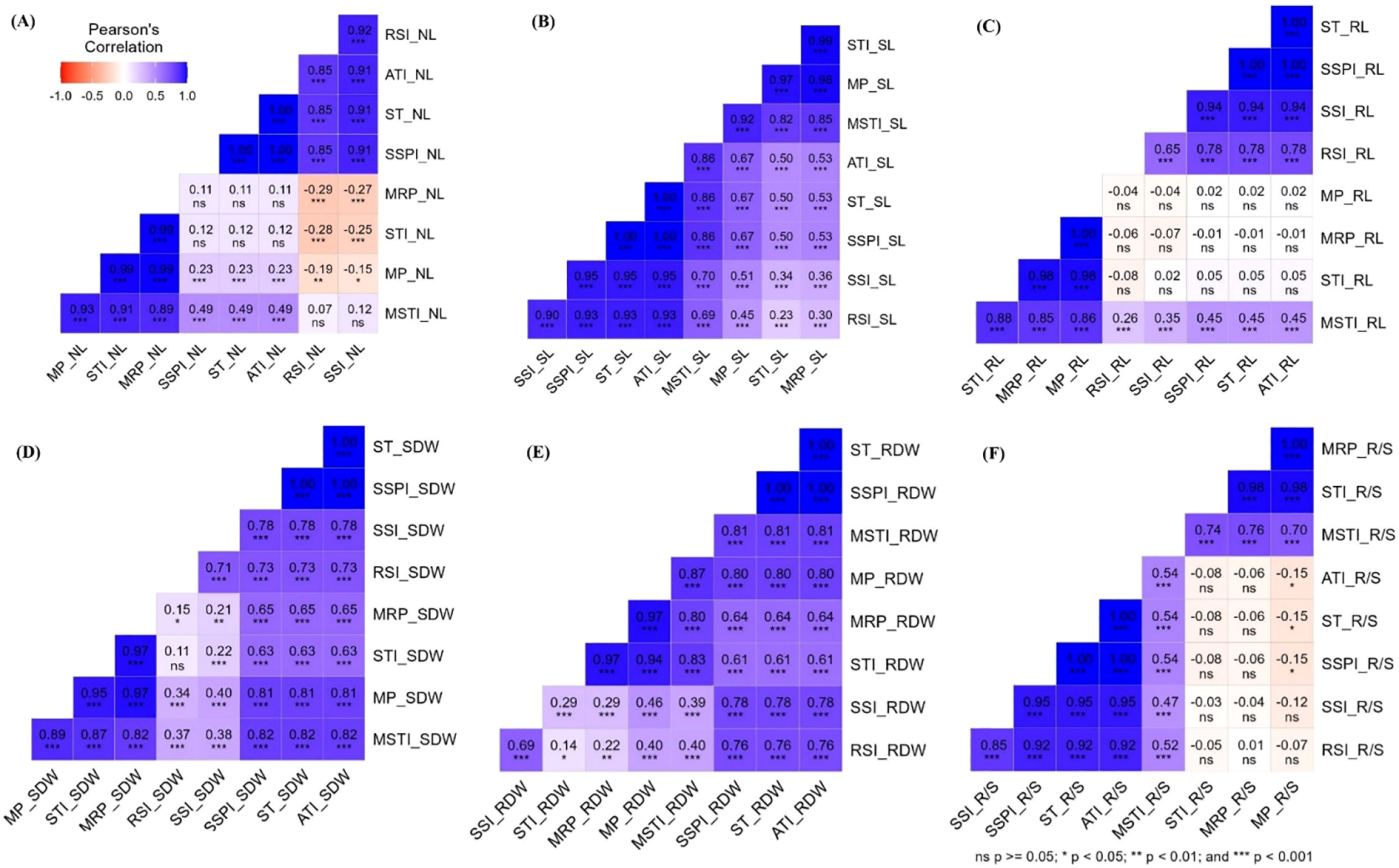
Pearson’s correlation coefficients among stress tolerance indices for the same trait. **(A)** Number of leaves (NL), **(B)** Shoot length (SL), **(C)** Root length (RL), **(D)** Shoot biomass (SDW), **(E)** Root biomass (RDW), and **(F)** Root-to-shoot ratio (R/S). ST, Stress tolerance; STI, Stress tolerance index; RSI, Relative stress index; MRP, Mean relative performance; SSI, Stress susceptibility index; MP, Mean productivity; ATI, Abiotic tolerance index; SSPI, Stress susceptibility percentage index; MSTI, Modified stress tolerance index. *p < 0.05; **p < 0.01; ***p < 0.001.

Pearson’s correlation analysis revealed a consistent relationship among seedling traits across both experiments (**Figure 2**). NL was significantly and positively correlated with SL, SDW, and RDW with correlation coefficients of 0.55, 0.77, and 0.68, respectively, and showed a weak negative correlation with R/S (-0.02) (**Figure 2**). The correlation between RDW and NL was similar in both chilling treatments (E2 and E3). SL was also positively correlated with SDW (0.81) and RDW (0.48), whereas SDW was positively correlated with RDW (0.78) and negatively correlated with R/S (-0.17). Notably, several trait-pair correlations (RDW-NL; R/S-SL; R/S-RL; RDW-SDW) were comparable between the two chilling treatments (E2 and E3), indicating consistent trait relationships under chilling conditions (**Figure 2**). To evaluate the relationship among stress tolerance indices (STIs), their corresponding trait values were extracted and examined. For NL, a significant negative correlation was observed between SSI and MRP, STI, and MP, and a similar pattern was observed with RSI. In contrast, a strong positive correlation was observed among STI, MP, MRP, and MSTI. For RL, a negative, non-significant correlation was found between RSI, SSI, SSPI, ST, and ATI, and MP, MRP, and STI. A similar pattern was observed for R/S, where ATI, ST, SSPI, SSI, and RSI showed negative, non-significant correlations with STI, MRP, and MP. These results indicate two distinct correlation groups among STIs for the same trait, set I (MRP = MP = STI ~ MSTI) and set II (RSI = ATI = SSI = ST = SSPI) (**Figure 3**).

### 3.3 Marker distribution, principal components, and kinship analysis

The SNPs spanned a total genome size of 372.9 Mb, with the highest number (102,787) covering 43.3 Mb on chromosome 1 and the lowest (55,380) spanning 29.9 Mb on chromosome 5 (**Supplementary Table 4**). Principal component analysis (PCA) revealed three subpopulations within the panel, collectively explaining 45.97% of the total genetic variation (**Supplementary Figure 3A**). The scree plot (**Supplementary Figure 3B**) showed that the first principal component (PC1) accounted for the largest proportion of variation (36.65%), followed by PC2 (6.79%) and PC3 (2.53%), indicating substantial genetic diversity within the panel. VanRaden kinship analysis showed that pairwise relatedness among accessions in the association panel was generally low, with kinship coefficients below 0.5, suggesting limited familial relatedness among the rice accessions (**Supplementary Figure 3C**). The low level of relatedness within the panel is advantageous for GWAS, as it reduces the false positives and increases the precision of marker-trait association.

### 3.4 Marker-trait associations for seedling stage chilling tolerance

#### 3.4.1 GWAS for seedling traits in Experiment 1

**Figure 4:**
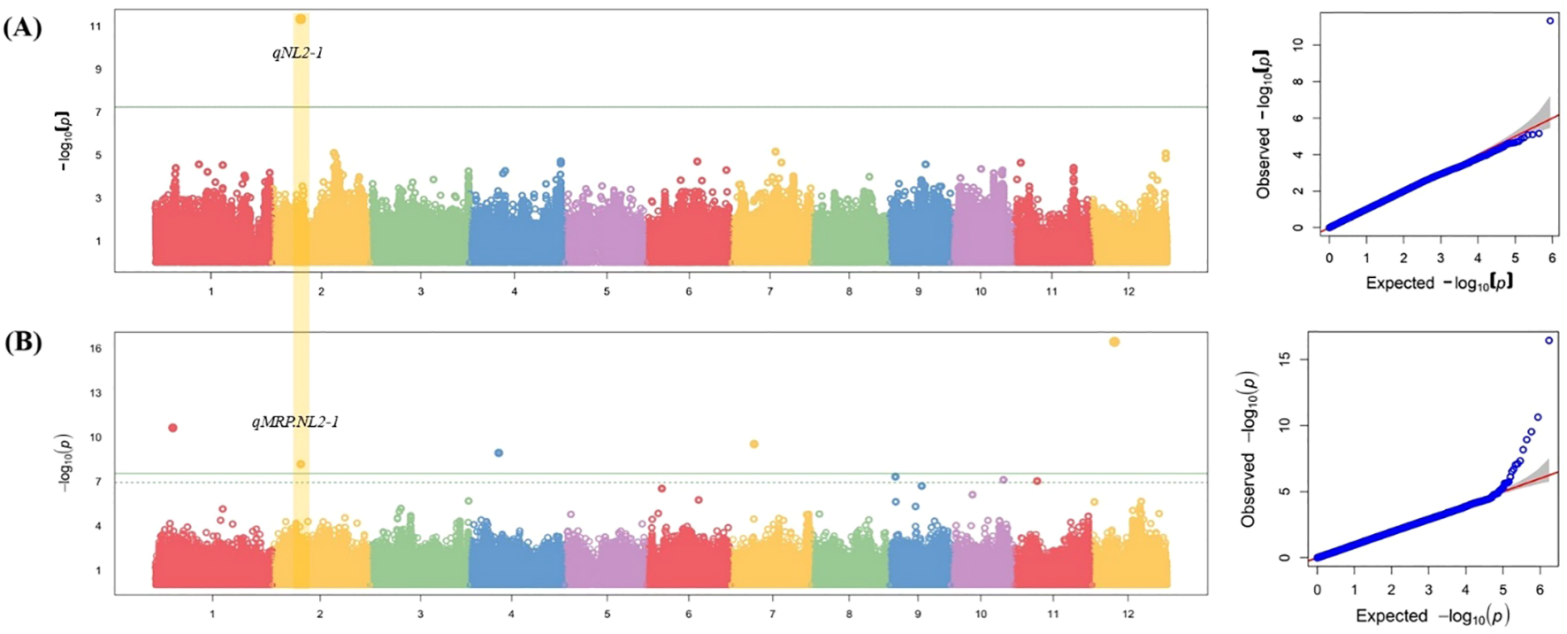
Manhattan and Q-Q plots showing the same significant SNP identified for the number of leaves. **(A)** *qNL2–1* in Experiment 1, and **(B)** locus for mean relative performance stress tolerance index, *qMRP.NL2–1* on chromosome 2. The x-axis indicates the number of chromosomes, and the y-axis indicates -log10(p) value. The solid green line represents the genome-wide significance threshold, and the dotted line indicates the suggestive threshold.

A total of ten QTLs were identified with a -log_10_ (*p*) value of ≥ 7.94, and these QTLs were detected by at least three of the four GWAS models in the E2 environment (**Table 2**). Among the ten QTLs, seven were co-localized with previously reported QTLs associated with seedling traits, supporting the reliability of the associations. The PVE by these QTLs ranged from 8.3% (*qSDW12-2*) to 50.7% (*qSDW2-2*). Under chilling (E2), three novel QTLs were identified: *qR.S1–1* for R/S and *qRDW1–1* for RDW on chromosome 1, and *qNL2–1* for NL on chromosome 2 (**Figure 4**). These novel QTLs explained between 8% and 42% of the phenotypic variance. Additionally, two QTLs on chromosome 1 (*qRS1–1* and *qRDW1-1*) and two QTLs on chromosome 12 (*qRDW12–1* and *qSDW12-2*) were located in close proximity, with physical distances of 34kb and 131kb, respectively. The co-localization of these loci suggests the robustness of the GWAS results, while the remaining loci likely represent novel QTLs for chilling stress tolerance identified in this study. However, no significant QTLs were detected for SL and RL under E2 conditions.

**Table 2:** List of QTLs identified by GWAS for chilling tolerance in Experiment 1 (Environment 1 and Environment 2).

Trait	SNP	Chr	QTL	GWAS model	-Log10(p)	PVE (%)	Known QTLs	Reference
R/S	S1_37992830	1	*qR/S1-1*	BLINK, FarmCPU, MLM	8.53	8.6	–	–
RDW	S1_37958048	1	*qRDW1-1*	BLINK, FarmCPU, MLM, GLM	8.62	15.5	–	–
NL	S2_10398058	2	*qNL2-1*	BLINK, MLM, GLM	11.33	42.1	–	–
SDW	S2_4212647	2	*qSDW2-2*	BLINK, MLM, GLM	13.58	50.7	*qSR2–2*	Li et al., 2025
R/S	S4_34903572	4	*qR/S4-1*	BLINK, FarmCPU, MLM, GLM	13.44	44.5	*qLTSS4-3*; *qLTS4*; *qMT4-3*	Schläppi et al., 2017; Shimoyama et al., 2020
SDW	S5_5767434	5	*qSDW5-2*	FarmCPU, MLM, GLM	8.74	12.9	*qLTG-5-1*	Jiang et al., 2006
SDW	S9_17341124	9	*qSDW9-1*	FarmCPU, MLM, GLM	10.05	10.0	*cisc(t)*	Lan et al., 2010
R/S	S11_6316909	11	*qR/S11-1*	FarmCPU, MLM, GLM	7.49	11.6	*dc11*; *fer11*	Oh et al., 2004
RDW	S12_3021933	12	*qRDW12-1*	BLINK, FarmCPU, MLM, GLM	8.62	27.9	*qMT12*; *qLT12; L121.ELC2*	Onishi et al., 2007; Lv et al., 2016; Shimoyama et al., 2020
SDW	S12_2890889	12	*qSDW12-2*	FarmCPU, MLM, GLM	8.53	8.3	*qMT12*; *L121.ELC2*	Onishi et al., 2007; Shimoyama et al., 2020

SNP, Single Nucleotide Polymorphism; Chr, Chromosome; QTL, Quantitative Trait Loci; GLM, General Linear Model; MLM, Mixed Linear Model; FarmCPU, Fixed and random model Circulating Probability Unification; BLINK, Bayesian-information and Linkage-disequilibrium Iteratively Nested Keyway; PVE, Phenotypic Variance Explained; R/S, Root-to-shoot ratio; RDW, Root biomass; RL, Root length; SDW, Shoot biomass; SL, Shoot length; and NL, Number of leaves.

#### 3.4.2 GWAS for seedling traits in Experiment 2

**Figure 5:**
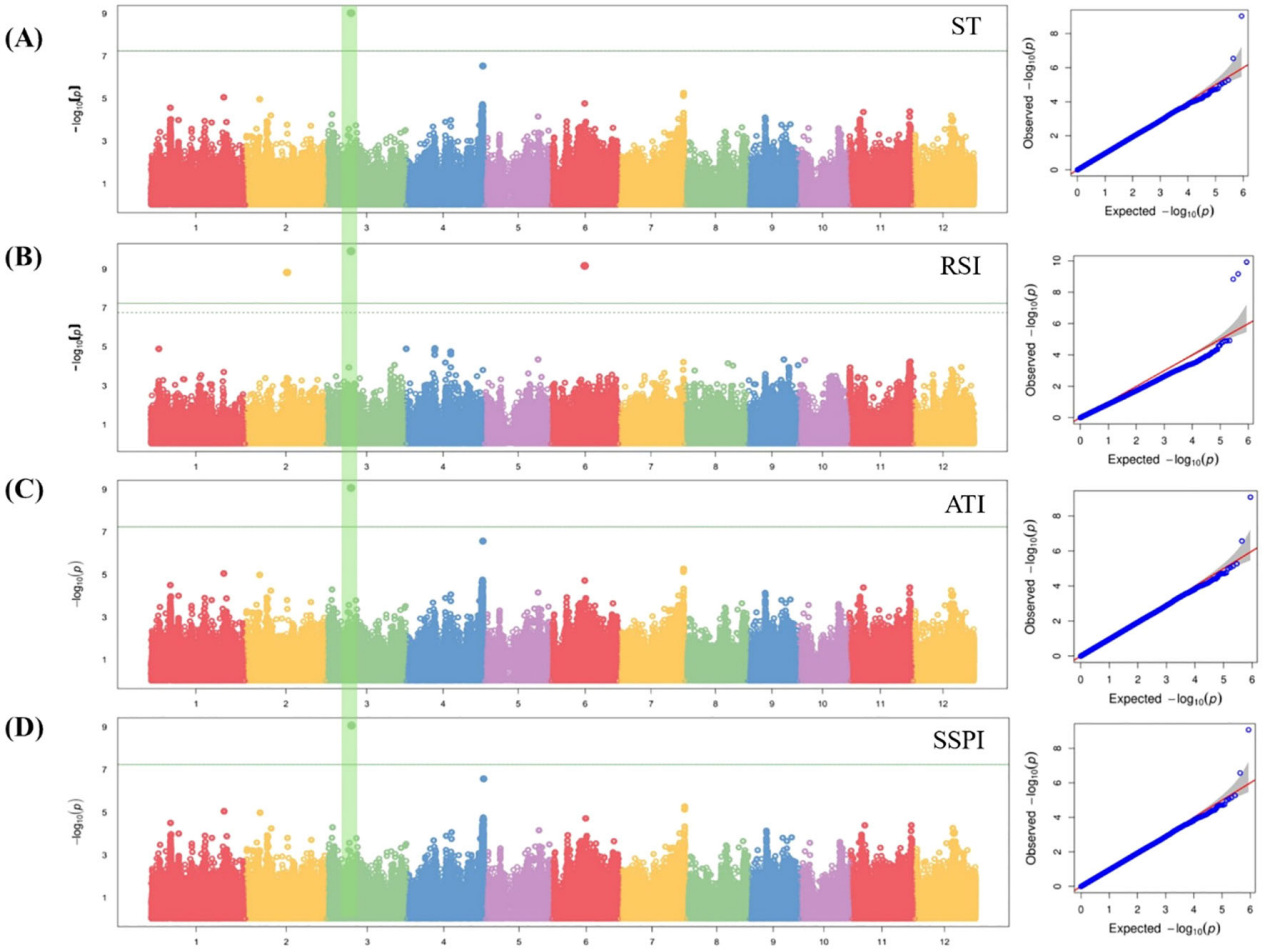
Manhattan and Q-Q plots for multiple stress tolerance indices for root-to-shoot ratio (R/S). **(A)** Stress tolerance (ST), **(B)** Relative stress index (RSI), **(C)** Abiotic tolerance index (ATI) and **(D)** Stress susceptibility percentage index (SSPI) indices using the BLINK algorithm.

In Experiment 2, none of the SNPs surpassed the stringent Bonferroni threshold of −log_10_ (*p*) = 7.94; therefore, the threshold was relaxed to −log_10_ (*p*) =5.0. Using this less stringent criterion, a total of 23 QTLs were identified (**Figure 5**; **Table 3**). Among these, six QTLs were co-localized with previously reported loci, while the remaining QTLs were considered novel. Chromosome 1 harbored the highest number of QTLs (eight), including one overlapping QTL, *qNC.R.S1-1*, associated with R/S, which co-localized with the previously reported locus *qrswca1*. Chromosome 4 contained four novel QTLs, whereas chromosome 9 had three novel and one overlapping QTL, *qNC.RDW9–1* is associated with RDW, which co-localizes with the reported *qCTGERM9-1*. Chromosomes 5 and 7 each contained two QTLs; among these, *qNC.RDW5–1* of chromosome 5, associated with RDW, overlapped with four previously identified QTLs (*qLTG-5-2*, *qCTS5*, *Locus 55* [*RLC*], and *qCTGERM5-1*. One QTL, *qNC.RDW2-1*, identified for RDW on chromosome 2 overlapped with previously reported *qPLR-2*, *qnob-5*, and *qSWTPNCT2-2*. Similarly, a single QTL on chromosome 11, *qNC.RDW11-1*, co-localized with three previously identified QTLs (*qLTG-11-1*, *qSCT11* and *Locus 114* [*ELSR2*]*)* (**Table 3**).

**Table 3:** List of QTLs identified by GWAS in Experiment 2 (Environment 3).

Trait	SNP	Chr	QTL	-Log10(p)	GWAS model	Known QTL/genes	Reference
R/S	S1_36081860	1	*qNC.R/S1-1*	10.44	BLINK, FarmCPU, GLM, MLM	*qrswca1*	Li et al., 2022
RDW	S1_38605808	1	*qNC.RDW1-1*	5.11	BLINK, FarmCPU, GLM, MLM	–	–
RL	S1_9875848	1	*qNC.RL1-1*	5.27	BLINK, FarmCPU, GLM, MLM	–	–
SDW	S1_34609357	1	*qNC.SDW1-1*	5.02	BLINK, FarmCPU, GLM, MLM	–	–
SL	S1_26010052	1	*qNC.SL1-1*	5.47	BLINK, FarmCPU, MLM	–	–
SL	S1_35060392	1	*qNC.SL1-2*	5.31	BLINK, FarmCPU, MLM	–	–
RDW	S2_26165469	2	*qNC.RDW2-1*	5.94	BLINK, FarmCPU, GLM, MLM	*qPLR-2*; *qSWTPNCT2-2*; *qnob-5*	Shakiba et al., 2017; Thapa et al., 2020
NL	S4_31681083	4	*qNC.NL4-1*	5.99	BLINK, FarmCPU, GLM, MLM	–	–
RDW	S4_2914775	4	*qNC.RDW4-1*	5.25	BLINK, FarmCPU, GLM, MLM	–	–
RDW	S4_2533441	4	*qNC.RDW4-2*	5.03	BLINK, FarmCPU, GLM, MLM	–	–
RL	S4_6233653	4	*qNC.RL4-1*	5.05	BLINK, FarmCPU, GLM, MLM	–	–
NL	S5_6372012	5	*qNC.NL5-1*	5.10	BLINK, FarmCPU	*qMT5-2*; *qLTS5*; *qCTS5-3*	Wang et al., 2016; Schläppi et al., 2017; Shimoyama et al., 2020
RDW	S5_21549721	5	*qNC.RDW5-1*	5.29	BLINK, FarmCPU, GLM, MLM	*qLTG-5-2*; *qCTS5*; *Locus 55 (RLC)*; *qCTGERM5-1*	Jiang et al., 2006; Li et al., 2013; Lv et al., 2016; Shakiba et al., 2017
NL	S7_28376132	7	*qNC.NL7-1*	5.41	BLINK, FarmCPU, MLM	*qLTG7*	Schläppi et al., 2017
RDW	S7_2541522	7	*qNC.RDW7-1*	5.21	BLINK, FarmCPU, GLM, MLM	*qMT7-2*; *Locus 69*; *qLTSS7-1*	Lv et al., 2016; Schläppi et al., 2017; Shimoyama et al., 2020
RL	S8_277636	8	*qNC.RL8-1*	5.49	BLINK, FarmCPU, GLM, MLM	–	–
NL	S9_7139081	9	*qNC.NL9-1*	5.64	BLINK, FarmCPU, GLM, MLM	*qMT9-3*; *qPGCG9-1*	Schläppi et al., 2017; Shimoyama et al., 2020
RDW	S9_10773791	9	*qNC.RDW9-1*	5.71	BLINK, FarmCPU, GLM, MLM	*qCTGERM9-1*	Shakiba et al., 2017
SDW	S10_22607917	10	*qNC.SDW10-1*	5.01	BLINK, FarmCPU, GLM, MLM	–	–
SL	S10_8703645	10	*qNC.SL10-1*	5.09	BLINK, FarmCPU, MLM	–	–
RDW	S11_22419265	11	*qNC.RDW11-1*	5.39	BLINK, FarmCPU, GLM, MLM	*qLTG-11-1*; *qSCT11*; *Locus 114; (ELSR2)*	Jiang et al., 2006; Kim et al., 2014; Lv et al., 2016
SDW	S12_24021515	12	*qNC.SDW12-1*	5.10	BLINK, FarmCPU, GLM, MLM	–	–
SL	S12_4731080	12	*qNC.SL12-1*	5.52	BLINK, FarmCPU, MLM	–	–

SNP, Single Nucleotide Polymorphism; Chr, Chromosome; QTL, Quantitative Trait Loci; GLM, General Linear Model; MLM, Mixed Linear Model; FarmCPU, Fixed and random model Circulating Probability Unification; BLINK, Bayesian-information and Linkage-disequilibrium Iteratively Nested Keyway; PVE, Phenotypic Variance Explained; R/S, Root-to-shoot ratio; RDW, Root biomass; RL, Root length; SDW, Shoot biomass; SL, Shoot length; and NL, Number of leaves.

#### 3.4.3 GWAS for STIs

**Figure 6:**
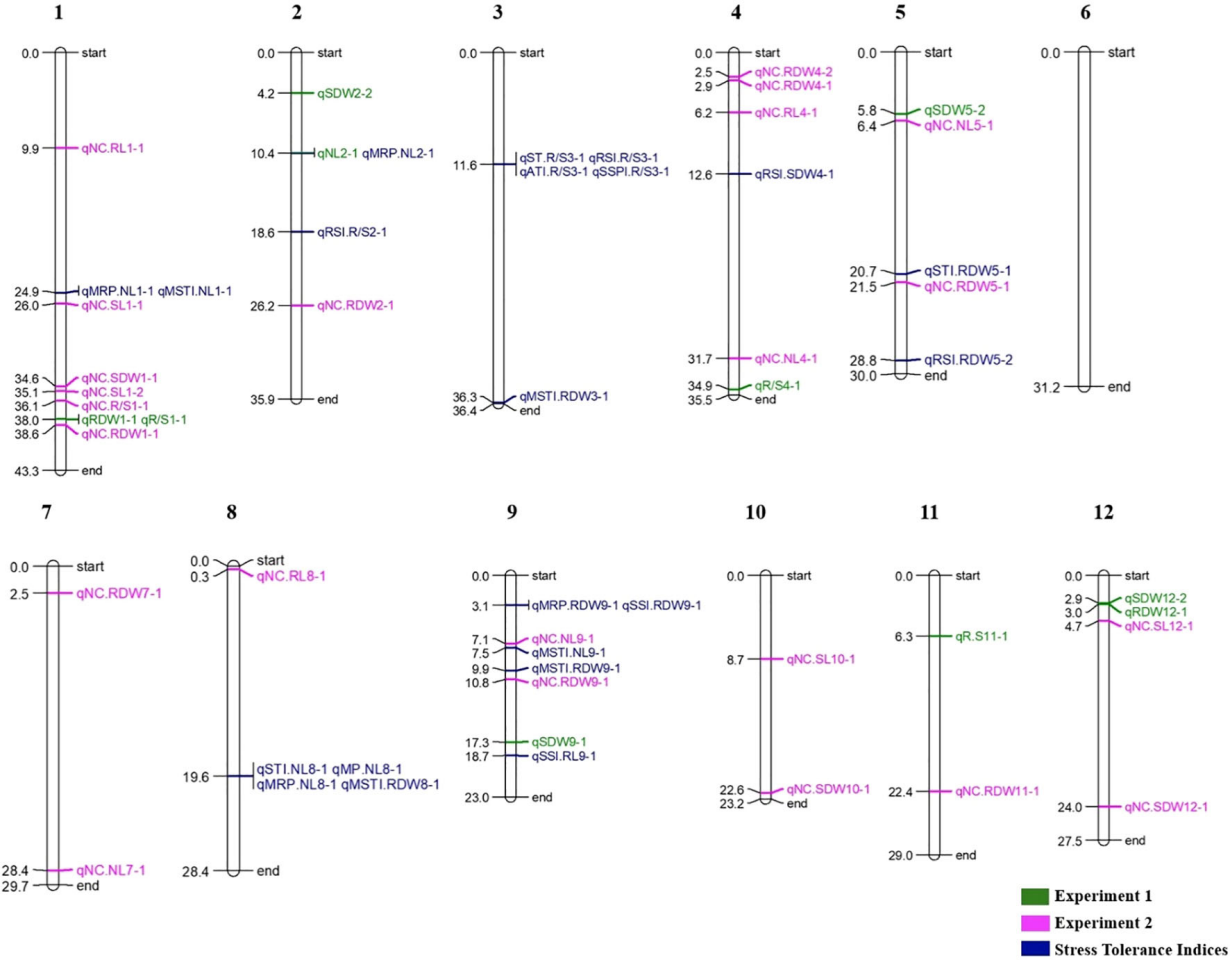
Comprehensive physical map showing the list of loci identified by GWAS for Experiment 1, Experiment 2, and Stress Tolerance Indices (STIs). Numbers on the left side indicate the position of the QTLs in megabase.

A total of nine STIs were calculated using seedling traits from Experiment 1, and GWAS was conducted independently for each index. Using a significance threshold of −log_10_ (*p*) = 7.94, we identified 21 QTLs, explaining between 5.3 to 82.2% of the PVE (**Table 4**). Among these QTLs, seven were associated with RDW and NL, five with R/S, and one each with SDW and RL. Among the RDW-associated QTLs, three (*qMRP.RDW9-1*, *qSSI.RDW9-1*, and *qMSTI.RDW9-1*) overlapped with the previously reported QTL, *clr9* on chromosome 9, while one QTL (*qRSI.RDW5-2*) co-localized with the reported QTL of *qGI-5–3* on chromosome 5. The remaining three RDW-associated QTLs were considered to be novel (**Table 4**). For NL, two QTLs (*qMRP.NL1–1* and *qMSTI.NL1-1)* co-localized with the reported gene, *Os01t0620100* on chromosome 1 (**Figure 6**; **Table 4**), while *qMSTI.NL9–1* overlapped with the previously reported QTLs of *clr9* and *qSR9* on chromosome 9. Five QTLs were identified for R/S, of which four QTLs (*qST.R.S3-1*, *qRSI.R.S3-1*, *qATI.R.S3-1*, and *qSSPI.R.S3-1)* were associated with a single SNP (S3_11628167) on chromosome 3 and overlapped with previously reported *qLVG3I*, *L34.BMR*, and *qSR3–3* (**Figure 6**; **Table 4**). In addition, a novel QTL, *qRSI.R.S2-1*, identified on chromosome 2, explained 15% PVE and showed no overlap with previous QTLs (**Table 4**). A single QTL for SDW (*qRSI.SDW4-1*), associated with RSI on chromosome 4, exhibited the highest PVE (82.2%) among all detected QTLs. For RL, one QTL (*qSSI.RL9-1*), associated with SSI, was identified on chromosome 9 and overlapped with three previously reported QTLs: *ELSR1/ELN*, *qPSR9*, and *cisc(t).*

**Table 4:** List of QTLs identified by GWAS for different stress tolerance indices (STIs).

STIs	SNP	Chr	QTL	GWAS model	-Log10(p)	PVE (%)	Known QTL/genes	Reference
MRP_NL	S1_24903854	1	*qMRP.NL1-1*	BLINK, FarmCPU	9.48	20.23	*Os01t0620100*	Song et al., 2018
MSTI_NL			*qMSTI.NL1-1*					
MRP_NL	S2_10398058	2	*qMRP.NL2-1*	BLINK, FarmCPU	8.14	39.86	–	–
RSI_R/S	S2_18604302	2	*qRSI.R/S2-1*	BLINK, FarmCPU	8.83	15.69	–	–
MSTI_RDW	S3_36325755	3	*qMSTI.RDW3-1*	BLINK, FarmCPU, MLM, GLM	14.51	17.70	–	–
ST_R/S	S3_11628167	3	*qST.R/S3-1*	BLINK, FarmCPU	9.02	42.13	*qLVG3I*; *L34.BMR*; *qSR3–3*	Han et al., 2006; Lv et al., 2016; Li et al., 2021
RSI_R/S			*qRSI.R/S3-1*					
ATI_R/S			*qATI.R/S3-1*					
SSPI_R/S			*qSSPI.R/S3-1*					
RSI_SDW	S4_12599143	4	*qRSI.SDW4-1*	BLINK, FarmCPU	11.46	82.18	–	–
STI_RDW	S5_20708232	5	*qSTI.RDW5-1*	BLINK, FarmCPU, MLM, GLM	8.34	72.49	–	–
RSI_RDW	S5_28837603	5	*qRSI.RDW5-2*	BLINK, FarmCPU, MLM	12.17	40.54	*qMT5-3*; *qGI-5-3; Locus 56*	Lv et al., 2016; Thapa et al., 2020; Shimoyama et al., 2020
STI_NL	S8_19626628	8	*qSTI.NL8-1*	BLINK, FarmCPU, GLM	11.42	41.61	–	–
MP_NL			*qMP.NL8-1*					
MRP_NL			*qMRP.NL8-1*					
MSTI_RDW	S8_19627711	8	*qMSTI.RDW8-1*	BLINK, FarmCPU, GLM	15.80	34.69	–	–
MSTI_NL	S9_7519938	9	*qMSTI.NL9-1*	BLINK, GLM	8.43	37.99	*qMT9-3*; *clr9*; *qSR9*; *qPGCG9–1*	Oh et al., 2004; Lan et al., 2010; Lv et al., 2016; Xiao et al., 2018; Li et al., 2021; Schläppi et al., 2017; Shimoyama et al., 2020
SSI_RL	S9_18729901	9	*qSSI.RL9-1*	BLINK, FarmCPU	11.80	62.23	*ELSR1/ELN*; *qPSR9*; *cisc(t)*	Lv et al., 2016; Xiao et al., 2018; Lan et al., 2007
MRP_RDW	S9_3149857	9	*qMRP.RDW9-1*	BLINK, FarmCPU	8.30	9.95	*qMT9-1*; *Locus 91*; *qCTS9-3*; *qCTS9-4*; *qCTS9-5*; *clr9*	Lv et al., 2016; Oh et al., 2004; Shimoyama et al., 2020; Wang et al., 2016
SSI_RDW			*qSSI.RDW9-1*					
MSTI_RDW	S9_9926920	9	*qMSTI.RDW9-1*	BLINK, FarmCPU	11.11	5.30	*clr9*	Oh et al., 2004

SNP, Single Nucleotide Polymorphism; Chr, Chromosome; QTL, Quantitative Trait Loci; GLM, General Linear Model; MLM, Mixed Linear Model; FarmCPU, Fixed and random model Circulating Probability Unification; BLINK, Bayesian-information and Linkage-disequilibrium Iteratively Nested Keyway; PVE, Phenotypic Variance Explained; STIs, Stress Tolerance Indices; NL, Number of Leaves; R/S, Root-to-shoot ratio; RDW, Root biomass; SDW, Shoot biomass; RL, Root length; SDW, Shoot biomass; MRP, Mean relative performance; MSTI, Modified stress tolerance index; RSI, Relative stress index; ST, Stress tolerance; ATI, Abiotic tolerance index; SSPI, Stress susceptibility percentage index; STI, Stress tolerance index; MP, Mean productivity; and SSI, Stress susceptibility index.

#### 3.4.4 Overlapping QTLs among Experiment 1, Experiment 2 and STIs

**Figure 7:**
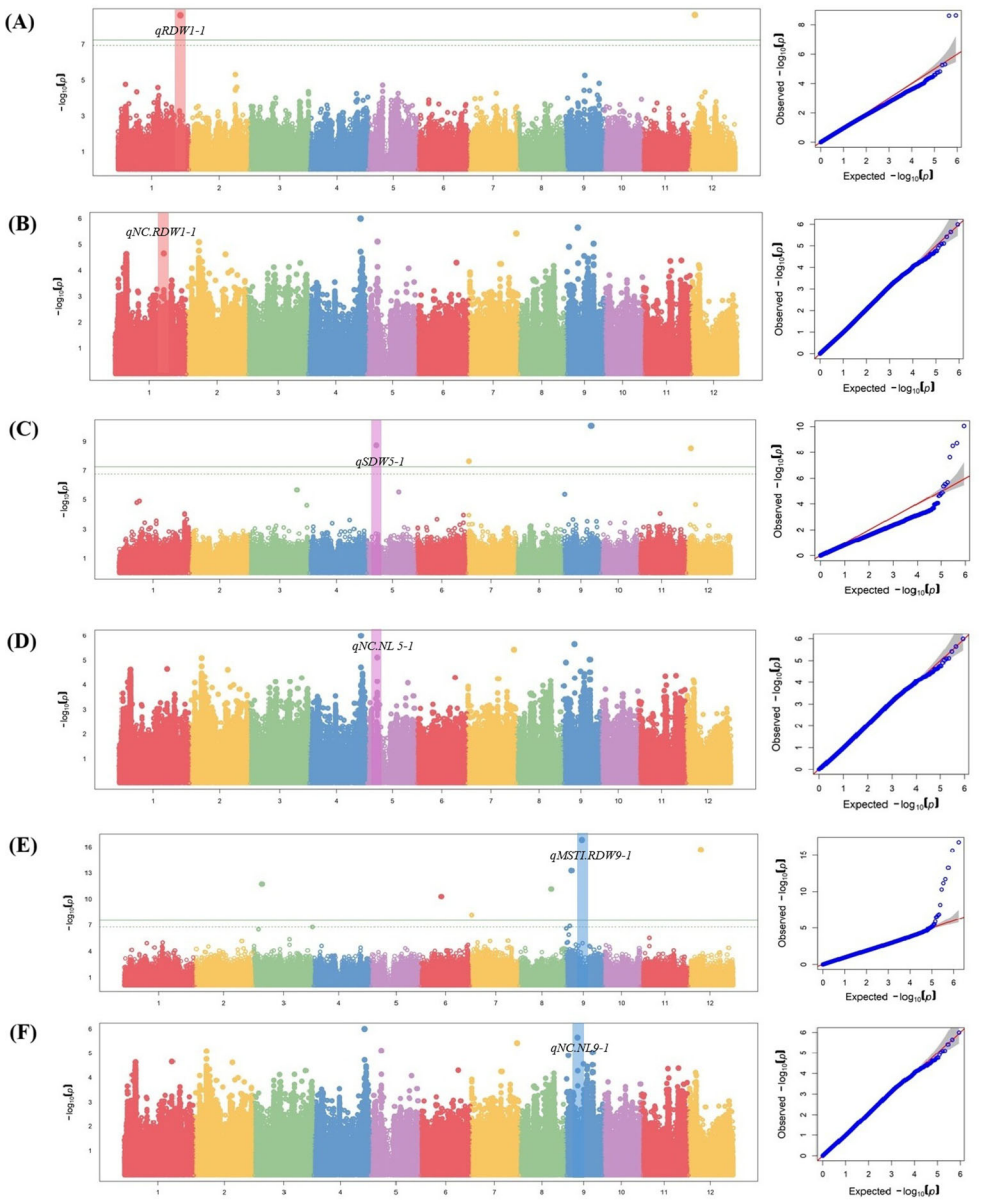
Manhattan and Q-Q plots for different traits across the experiments. **(A)** *qRDW1-1* (Exp. 1, root dry weight) and **(B)** *qNC.RDW1-1* (Exp. 2) in chromosome 1. **(C)** *qSDW5-1* (Exp. 1) and **(D)** *qNC.NL 5-1* (Exp. 2) in chromosome 5. **(E)** *qMSTI.RDW9-1* (Modified stress tolerance index for root dry weight) and **(F)** *qNC.NL9-1* (Exp. 2) in chromosome 9. RDW, root dry weight; SDW, shoot dry weight; and NL, number of leaves.

A total of 54 QTLs were identified across the independent GWAS analyses of Experiment 1, Experiment 2 and the STIs. Notably, one QTL from E2 (*qNL2-1*) and another from STI analysis (*qMRP.NL2-1*) mapped to the same genomic position (S2_10398058) for NL, representing a region that has not been reported in earlier studies (**Figures 4**, **6**). Apart from this region, no other identical QTLs were detected across the three datasets; however, several loci are in LD. For example, two QTLs identified in E2, *qRDW1-1* (RDW) and *qR.S1-1* (R/S), were separated by only 34.7 kb and were in LD with *qNC.RDW1-1*, a QTL for RDW identified in E3. Similarly, *qSDW5-2* (SDW), identified in E2, was in LD with *qNC.NL5-1* (NL) in E3. On chromosome 9, *qMSTI.RDW9-1* (from STI analysis) and *qNC.NL9-1* (from E3) also showed in LD, suggesting a potentially shared region influencing multiple traits under chilling stress (**Figure 7**).

### 3.5 Putative candidate gene identification

**Figure 8:**
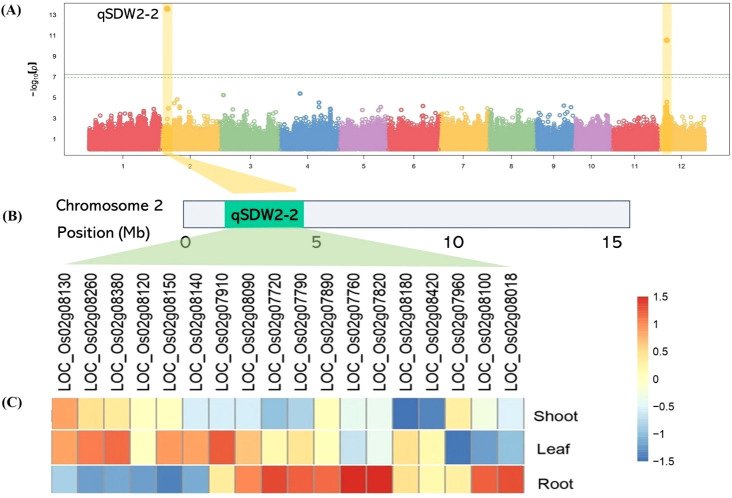
Strategy used to identify putative candidate genes in GWAS-detected QTLs. **(A)** Manhattan plot showing the significant SNP above the genome-wide significance threshold. **(B)** Location of QTL on the respective chromosome. **(C)** Heatmap showing the mean FPKM (Fragments Per Kilobase of transcript per Million mapped reads) values of candidate genes across different tissues under chilling stress, derived from Plant Public RNA-seq Database (https://plantrnadb.com/ricerna/). This database contains FPKM values from more than 10000 publicly available RNA−seq libraries derived from multiple independent studies and are processed using a uniform pipeline. Each value represents the average expression across multiple independently generated RNA−seq samples annotated under similar conditions. Expression values are normalized FPKM and are not log_2_ fold changes relative to control. The RNA−seq libraries included in the database represent a mixture of rice accessions from different genetic backgrounds.

To identify candidate genes within the QTL regions, a 670-kb genomic region centered on each significant SNP was examined using the Nipponbare reference genome in the Rice Annotation Project Database (RAP-DB) (**Figure 8**). Genes annotated as hypothetical or of unknown function were excluded from further analysis. Putative candidate genes were then evaluated for their expression in leaves, stems, and roots under chilling stress using the Plant Public RNA-seq Database (https://plantrnadb.com/ricerna/). Based on this analysis, a total of 86, 72, and 75 putative candidate genes with known functions related to chilling stress were identified from Experiments 1, 2 and the STI-based analyses, respectively (**Supplementary Tables 5, S6, S7**). Notable candidates included genes involved in calcium signaling, transcriptional regulation and stress response pathways, such as *LOC_Os12g06570* (CNGC 8), *LOC_Os12g06100* (EF-hand type motif), *LOC_Os11g11340* (calmodulin-interacting protein), and *LOC_Os09g28440* (AP2 domain protein), *LOC_Os02g08120* (calmodulin-binding protein), *LOC_Os02g08018* (calcium-transporting ATPase), *LOC_Os01g64960* (chlorophyll a-b binding protein), *LOC_Os01g43650* (WRKY transcription factor), *LOC_Os08g31980* (Trehalose-6-phosphate synthase), *LOC_Os09g31390* (bZIP transcription factor), and *LOC_Os09g15790* (GTP-binding protein).

## 4 Discussion

Chilling stress is becoming a significant challenge for rice production, particularly as farmers shift from traditional puddled transplanting to direct seeding to reduce labor and input costs. However, low germination rates and slow seedling growth in many modern rice cultivars limit the widespread adoption of direct seeded method. This study focused on identifying chilling-tolerant donors and loci for rice improvement.

### 4.1 Plasticity of rice association panel for chilling stress tolerance

In this study, we used a rice association panel to capture genetic variability and identify significant QTLs associated with chilling stress tolerance. Most studies investigating chilling stress have evaluated rice accessions under temperature-controlled growth chambers, which allow precise control of environmental factors and space. In many previous studies, rice seedlings were exposed to cold temperatures ranging from 10 to 14 °C for 7–10 days, followed by a recovery period of 2–7 days under control conditions (24 to 28 °C) (Li et al., 2022). However, such experimental designs do not adequately simulate natural chilling conditions, particularly the temperature fluctuations plants experience in the field. To address this limitation, we conducted Experiments 1 and 2 to evaluate seedling traits under low-temperature conditions. Significant phenotypic variation was observed among accessions for all traits across both experimental conditions (**Table 1**), indicating that the panel used in this study represents valuable genetic resources for chilling stress tolerance research. These findings are consistent with a previous study by Singh et al. (2025), who reported significant phenotypic variation in seed emergence and seedling length traits in *japonica* and *indica* panels evaluated under similar experimental setups. Compared with E1, chilling stress (E2) significantly reduced the mean values of SL, SDW, and RDW traits. Under the control condition (E1), most accessions germinated within 5–7 days, whereas under the natural chilling condition (E3), germination began 13 days after sowing and continued until 31 days, with an overall germination rate of 78%. These reductions may be attributed to disruptions in plant hormones such as abscisic acid (ABA) and gibberellic acid (GA_3_), which play critical roles in regulating germination, growth, and development. Similar declines in germination and early shoot and root growth traits have been reported at temperatures around 12°C during early-season rice cultivation (Zhang et al., 2020; Zhang et al., 2023). In contrast, the RL and R/S increased under chilling stress, suggesting a trade-off mechanism in response to low temperatures. Under E3 conditions, some accessions exhibited poor shoot growth but developed a profuse root system, which may have supported the uptake of essential nutrients and water, thereby helping to maintain metabolic activity. Additionally, reduced stomatal conductance and lower transpirational demand under chilling stress may promote greater allocation of carbohydrates to roots rather than the shoot in tolerant accessions. This redistribution of resources likely explains the increased root development and R/S observed under chilling conditions compared with the control (E1). Notably, several accessions (Antonio, RU1501007, Katy, and Maybelle) germinated earlier and outperformed the chilling-tolerant check (IRGC 32567) under E3 conditions, indicating their superior adaptability to chilling stress. Pearson correlation analysis further revealed that several trait correlations (RDW and NL; R/S and RL; RDW and SDW) were similar between both chilling treatments (E2 and E3), demonstrating the robustness and reliability of phenotypic evaluation (**Figure 2**).

### 4.2 Identification of the best stress-tolerant index for QTL discovery

Chilling stress is a complex genetic trait, involving many genes with diverse effects that depend on developmental stage and environmental conditions (Li et al., 2022). Employing both direct and indirect phenotypes to capture the genetic loci underlying chilling tolerance may therefore yield more robust results than relying on either approach alone. Stress tolerance indices (STIs) mathematically integrate performance under stress and non-stress conditions and understanding the mechanisms underlying these indices provides valuable insights into stress tolerance (Tsehaye et al., 2024). Earlier studies using STIs focused on selecting superior accessions under stress or non-stress conditions, with only a few employing STIs for QTL discovery (Schläppi et al., 2017; Majidimehr et al., 2024). Although such approaches are more common in wheat (Qaseem et al., 2019; Rabieyan et al., 2023; Reddy et al., 2023; Majidimehr et al., 2024), relatively few studies have applied similar strategies in rice (Schläppi et al., 2017). In this study, we calculated 9 STIs using phenotypic data from Experiments 1 and 2 (E1 and E2) and performed GWAS to identify QTLs underlying chilling stress tolerance. An interesting correlation pattern was observed among the nine STIs, which were grouped into two sets: set I (MRP = MP = STI ~ MSTI) and set II (RSI = ATI = SSI = ST = SSPI) (**Figure 3**). Kalve et al. (2022) reported that MP and MSTI were positively correlated with seed yield under stress conditions but negatively correlated with tolerance indices such as TOL (equivalent to ST), ATI, and SSPI. Similarly, Majidimehr et al. (2024) reported strong correlations between STI and MP, and between SSI and TOL, but only a weak correlation between STI and TOL (r=0.19). Fernandez (1992) reported a positive correlation between STI and MP (r=0.88), and negative correlations between TOL and STI (r=-0.28) and between SSI and STI (r=-0.84). Consistent with these findings, our results indicate that STI and TOL (ST in this study) belong to separate sets and exhibit negative or non-significant correlations. Collectively, these studies support the distinct correlation patterns among stress indices observed in the present study. Based on these results, we recommend using at least one index from set 1 (MRP, MP, or STI) and one from set II (ST, SSPI, SSI, ATI, or RSI) for ranking the accessions or identifying QTLs for complex traits, as each set captures complementary information essential for elucidating the genetic architecture of stress tolerance.

### 4.3 Optimizing genetic loci discovery using stress tolerance indices

Correlation analysis revealed that the 9 STIs could be broadly separated into two functional groups. The Set I indices (MRP, MP, STI, and MSTI) focused on overall growth maintenance and productivity under both stress and non-stress conditions. These indices appear to be more suitable for identifying loci associated with vigor and stable performance across stress and non-stress environments. In contrast, set II indices (ST, SSPI, SSI, ATI, and RSI) were strongly associated with stress sensitivity and relative injury responses, reflecting that they better capture the magnitude of performance reduction due to stress. Based on these findings, we propose optimization strategies for stress-tolerance GWAS studies. First, when the research objective is to identify loci associated with stable growth performance, representative indices from set I, such as MRP or STI, may be prioritized, as they integrate performance under both conditions and reduce redundancy among highly correlated indices. Second, when the objective is to identify loci associated with stress susceptibility, injury response, or relative performance reduction due to stress, representative indices from set II, such as SSPI or ST, can be chosen. Third, because the two sets capture complementary information, combining at least one index from each set would likely improve QTL discovery and facilitate the dissection of the complex genetic architecture underlying stress tolerance. Our results suggest that selecting representative STIs improves statistical efficiency in GWAS by reducing multicollinearity and minimizing redundancy among highly correlated indices, as supported by QTL discovery across distinct correlation sets. For example, STI, MRP, and MP (set I) were associated with the S8_19626628 region for NL, while ST, RSI, ATI, and SSPI (set II) colocalized at the S3_11628167 region for R/S (**Figure 5**). Moreover, the co-localization of a QTL identified in E2 (*qNL2-1*) with *qMRP.NL2-1* (identified through STIs) at the same genomic location (S2_10398058) for NL (**Figures 4**, **6**) provides additional support for the distinct correlation patterns among STIs and their utility in QTL discovery.

### 4.4 Studies supporting *japonica*-based chilling tolerance

Tropical adapted species, including rice, are generally sensitive to chilling stress, and their geographic distribution is partly determined by this sensitivity (Morsy et al., 2005). Among rice subspecies, *indica* rice tends to be more susceptible to chilling than *japonica* rice (Cheng et al., 2007). Previous studies reported that the cold tolerance of *japonica* rice is partly attributable to genetic variation in the cold-responsive gene, *COLD1.* Specifically, the *COLD1* gene enhances cold tolerance in accessions carrying the *COLD1^jap^* allele compared with those carrying the *COLD1^ind^* allele (Ma et al., 2015). Supporting this observation, Pan et al. (2015) evaluated a mini-core collection of 174 Chinese rice accessions and reported greater cold tolerance in *japonica* rice than in *indica*. Similarly, Thapa et al. (2020) reported that the *japonica* rice group is generally more cold-tolerant than the *aus*, aromatic, and *indica* groups, likely because *indica* rice is better adapted to warmer, low-latitude regions, whereas *japonica* rice is adapted to colder, high-latitude regions and higher elevations. To understand this relationship, we separated *japonica* accessions from the panel and performed GWAS. At least half of the loci identified in the whole panel were detected in the *japonica*-based GWAS analysis (**Supplementary Table 8**), supporting the statement that *japonica* is superior to *indica* in terms of chilling stress tolerance. Association mapping has been widely applied to dissect complex traits important for agronomy and crop improvement in many staple crops (Shao et al., 2011). Our findings demonstrate that association mapping is more efficient than QTL detection for complex traits such as chilling stress tolerance. Previous studies have identified several important QTLs associated with chilling tolerance during germination and seedling stages, including *qCTS2*, *qCTS7*, *qLTG-3*, *qLTG-11-1*, and *clr9* (Lv et al., 2016). However, these QTLs often span large physical intervals, making fine mapping challenging. In addition, QTL mapping requires high-quality mapping populations, which are both time-consuming and resource-intensive (Holland, 2007). Nevertheless, successful fine-mapping efforts have been reported; for example, *qLTG-9* and *qLTG3–1* have been fine-mapped and characterized (Fujino et al., 2004; Li et al., 2013), and chilling tolerance genes such as *COLD1* have been successfully cloned (Ma et al., 2015).

### 4.5 Comparison with previous GWAS studies on rice chilling tolerance

Previous GWAS studies on rice chilling tolerance have employed diverse germplasm panels, generally ranging from approximately 117 to 421 accessions, including regional collections, diversity panels, mini-core collections, landraces, and subsets of the 3K rice genome project. For instance, Ham et al. (2021); Kim et al. (2021), and Jeong et al. (2021) evaluated Korean rice accessions under controlled cold treatments using tolerance scores, spikelet fertility, or recovery-based phenotypes and conducted GWAS using EMMA, MLM, or FarmCPU-based approaches. Larger diversity panels, such as those reported by Li et al. (2021); Li et al. (2022); Shakiba et al. (2017); Schläppi et al. (2017); Shimoyama et al. (2020); Song et al. (2018); Thapa et al. (2020), and Rastogi et al. (2025), further expanded population diversity by incorporating *indica-japonica* substructure and evaluating chilling tolerance at seedling or reproductive stages under controlled environments. Despite differences in population size, genetic background, and experimental design, a common feature among most previous studies is the reliance on limited phenotypic descriptors of chilling tolerance, such as survival rate, tolerance score, seedling vigor, spikelet fertility, or qualitative cold injury ratings. Although some studies (e.g., Shakiba et al., 2017; Schläppi et al., 2017; Rastogi et al., 2025) incorporated stress-derived indices. In addition, most previous studies primarily focused on above-ground traits, while root-related traits were comparatively underexplored despite their important roles in stress adaptation and resource acquisition under chilling conditions.

The present study integrated six morphological traits related to shoot growth, root development, biomass accumulation, and biomass allocation under both stress and non-stress conditions, as well as nine STIs. Moreover, chilling tolerance was evaluated under both controlled environment and natural chilling conditions, providing a broader representation of the genetic architecture underlying tolerance. Most previous studies have employed individual GWAS models, such as MLM, EMMA, FarmCPU, or EMMAX, to assess marker-trait associations. Although these models effectively account for population structure and kinship effects, individual models may differ in statistical power and false-positive control. To improve the robustness of locus detection, the present study employed multiple GWAS models, including GLM, MLM, FarmCPU, and BLINK, and considered loci as reliable only when consistently detected by at least two models. This stringent multi-model strategy, along with shoot and root traits, increased confidence in the identified associations under both controlled and natural chilling conditions and reduced the likelihood of model-specific false-positive signals.

### 4.6 LD pattern of rice association panel

Examining LD patterns has gained significant interest in rice research, both to understand population evolutionary dynamics and to facilitate gene discovery (Garris et al., 2003; Rakshit et al., 2007). Several factors contribute to the extent of LD, including selection, mutation, inbreeding, substructure, admixture, and genetic drift. However, the effective recombination rate is the primary force shaping LD, as recombination between loci progressively degrades LD over generations. Early studies reported that rapid LD decay (approximately 5 kb) in the wild progenitor *O. rufipogon*, likely due to higher recombination rates, whereas *indica* rice exhibits comparatively slower LD decay (50 kb) (Rakshit et al., 2007). In contrast, LD decay in *japonica* rice has been reported to extend beyond 500 kb (Mather et al., 2007), suggesting lower recombination rates than in *indica*. Variation in recombination rates across genomic regions has also been observed, likely due to recombination hotspots, as reported in humans (McVean et al., 2004). In the present study, we observed an LD decay distance of approximately 670 kb in a panel of 233 accessions using 872,995 SNPs. This relatively extended LD decay indicates substantial genetic diversity with moderate historical recombination rates, making this panel well-suited for association mapping of complex traits such as chilling stress tolerance in rice.

GWAS performed using STIs produced markedly higher PVE values (82.18% for *qRSI.SDW4–1* and 72.49% for *qSTI.RDW5-1*) than GWAS using actual control or stress data. This could be because STIs reduce environmental noise between treatments and isolate the genotype × environment interaction component, thereby reducing phenotypic variance, as seen in Experiment 1. The shoot biomass ranged from 13.50 to 126.58 g (E1, control) and 5.30 to 50.18 g (E2, chilling), whereas RSI (Relative Stress Index) ranged from 0.20 to 6.00. Similarly, root biomass ranged from 3.50 to 73.77 g (E1) and 1.60 to 29.60 g (E2), while STI ranged from 0.04 to 2.86. These phenotypic variance dispersions between the groups and STIs clearly show that STIs eliminate background noise, thereby reducing the total phenotypic variance. This change in phenotypic variance could be one of the reasons for the increase in PVE to a specific locus.

### 4.7 Calcium-based cell signaling for chilling stress tolerance

Calcium (Ca²^+^) functions as a critical second messenger in plant responses to cold stress (Dodd et al., 2010). Low temperatures trigger Ca²^+^ transients in both the cytosol and nucleus, which are mediated by calmodulin (CaM)/CaM-like (CML) proteins, calcineurin B-like (CBL) proteins, and calcium-dependent protein kinases (CDPKs) (Luan, 2009). In *Arabidopsis*, overexpression of the *CaM3* gene suppresses the expression of the cold-responsive gene *KIN1*, suggesting that *CaM3* may function as a negative regulator of cold-induced gene expression and modulate plant responses to low temperature tolerance (Townley and Knight, 2002). Beyond CDPKs and CIPK signaling, the mitogen-activated protein kinase (MAPK) cascade also plays a crucial role in cold stress signaling. Low temperatures activate MKK6 phosphorylation, which subsequently triggers MPK3/6 signaling, thereby enhancing cold tolerance in rice (Xie et al., 2014). Furthermore, rice MAPK3 phosphorylates the inducer of CBF expression 1 (*ICE1*), which activates the trehalose phosphatase gene (*TPP1*), leading to increased trehalose production and improved chilling tolerance (Zhang et al., 2017). Small GTP-binding proteins, particularly Rab-type proteins, also contribute to chilling tolerance pathways in rice (Chen et al., 2011). For example, *OsRAB7* and Ran-type small G-proteins (*OsRNA1* and *OsRNA2*) help maintain nuclear envelope integrity and regulate gene expression in response to chilling stress (Nahm et al., 2003; Chen et al., 2011). Similarly, genes such as *AtCML24/TCH2* and *OsMSR2*, which encode CML proteins, are induced by cold and likely transduce cold-induced Ca²^+^ signals (Xu et al., 2011). In the present study, the majority of candidate genes including *LOC_Os12g06510* (Calcineurin B-like protein 6), *LOC_Os12g06570* (Cyclic nucleotide-gated channel 8), *LOC_Os11g11340* (Calmodulin-interacting protein 111), *LOC_Os02g08120* (Calmodulin-binding protein 60-B), *LOC_Os02g08140* (Calcium/calmodulin-dependent protein kinases), *LOC_Os04g58570* (C2 calcium/lipid-binding region), and *LOC_Os02g08018* (Calcium-transporting ATPase 9) found to be associated with stress tolerance. These genes represent putative candidates encoding components of Ca²^+^/CaM-mediated signaling pathway involved in chilling stress tolerance.

## 5 Conclusion

In this study, we evaluated a rice association panel of 233 accessions for their tolerance to chilling stress during the seedling stage. We employed two independent experimental setups and analyzed nine different stress-tolerance indices (STIs). GWAS identified 54 QTLs, 28 of which were co-localized with QTLs previously reported in other studies. This study identified QTL, novel *qNL2-1*, which was consistently detected in E2 and the MRP stress tolerance index and has not been reported in earlier studies. Our findings highlight the importance of calcium-mediated signaling pathways in chilling stress tolerance. Overall, this study demonstrates the effectiveness of combining controlled-environmental experiments with natural chilling conditions and various stress-tolerance indices to accurately capture genetic variation. The identification of novel QTLs and putative candidate genes in this study provides valuable resources to help breeders enhance chilling stress tolerance in rice.

## Data Availability

The datasets presented in this article are not readily available because the genotypic dataset was generated by collaborators for an independent ongoing research project; the collaborators have yet to publish the full data. Requests to access the datasets should be directed to the corresponding author.
