# Supplementary material for: Genetic architecture of seedling stage chilling tolerance in United States rice germplasm
Source: Front Plant Sci. 2026 Jul 9;17:1845537. doi: 10.3389/fpls.2026.1845537 (PMC13391267; doi:10.3389/fpls.2026.1845537)
Supplement: Supplementary file 2 [file SupplementaryFile1.docx]

Supplementary Material


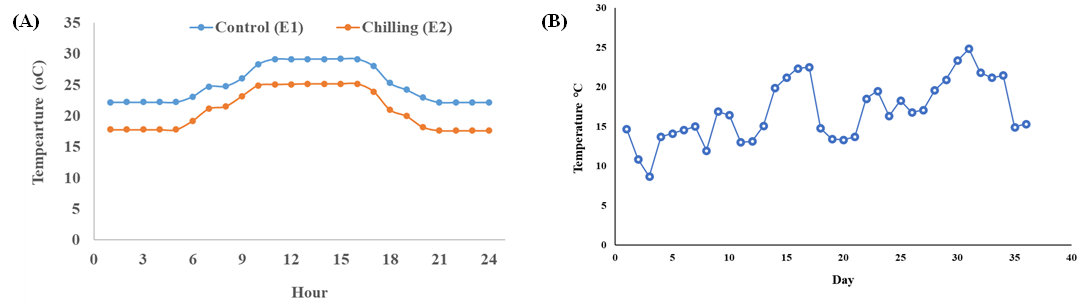


**Supplementary Figure 1.** Temperature profiles throughout the duration of **(A)** Experiment 1 [Environment 1 (E1): Control and Environment 2 (E2): Chilling] and **(B)** Experiment 2 [Environment 3 (E3): Natural chilling]. x-axis indicates the hour **(A)** and day **(B)**, while y-axis indicate temperature in degree celsius.


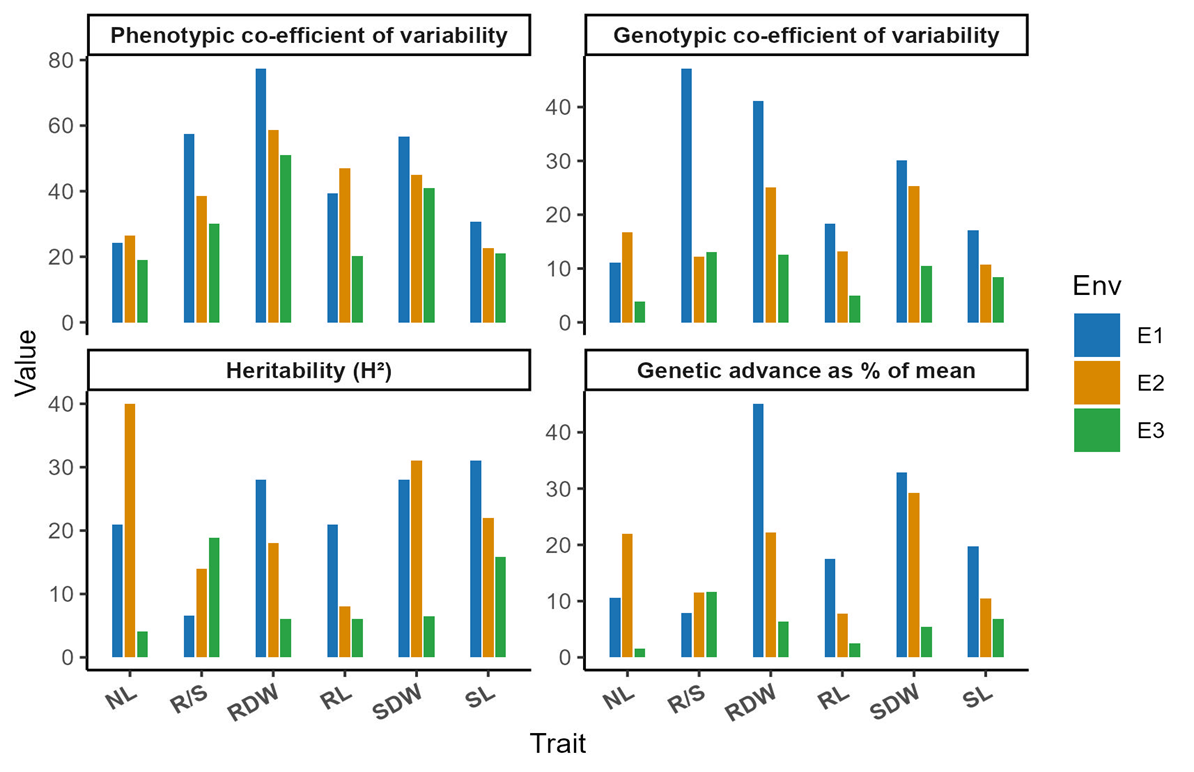


**Supplementary Figure 2.** Genetic variability parameters across three environments. Environment 1 (Control) (E1), Environment 2 (Chilling) (E2), and Environment 3 (Natural chilling) (E3). NL: Number of leaves. SL: Shoot length. RL: Root length. SDW: Shoot biomass. RDW: Root biomass. R/S: Root-to-shoot ratio. The x-axis indicates the trait name, and the y-axis indicates the genetic variability parameter value.


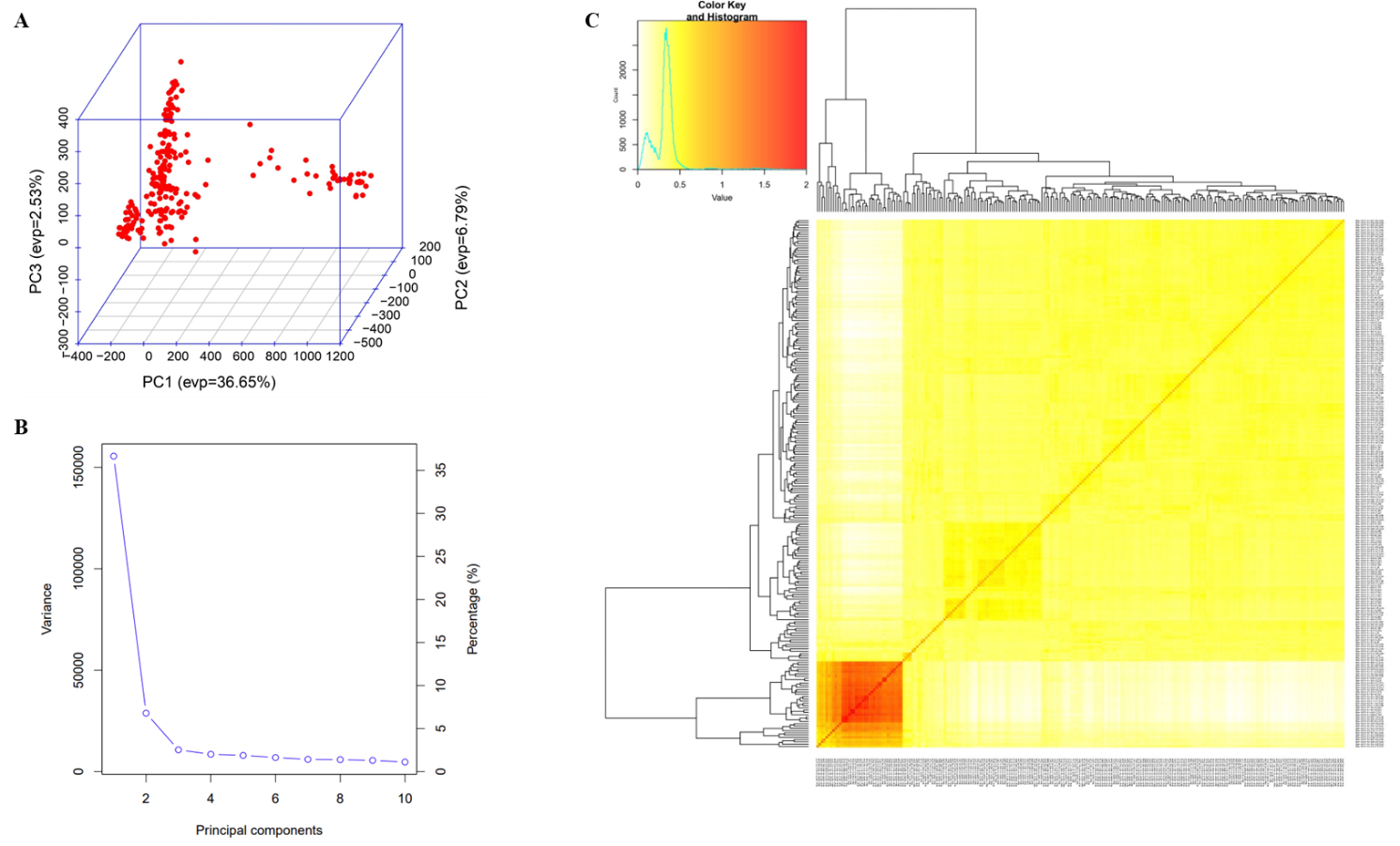


**Supplementary Figure 3.** Population structure analysis of 233 accessions in the rice association panel based on 8,72,995 SNPs. **(A)** 3D representation of principal component (PC) analysis showing three populations. **(B)** Scree plot depicting the number of significant PCs. There were three PCs that explained a cumulative variation of 45.97%. **(C)** Heatmap of the kinship matrix. The heatmap shows the level of relatedness among the population. The pattern of darker red areas showed a high level of relatedness between genotypes, and the dendrogram depicts the clustering of accessions.
